# Study of β_1_-transferrin and β_2_-transferrin using microprobe-capture in-emitter elution and high-resolution mass spectrometry

**DOI:** 10.1038/s41598-023-42064-7

**Published:** 2023-09-11

**Authors:** Ruben Yiqi Luo, Christopher Pfaffroth, Samuel Yang, Kevin Hoang, Priscilla S.-W. Yeung, James L. Zehnder, Run-Zhang Shi

**Affiliations:** 1 Department of Pathology, Stanford University, Stanford, CA USA; 2 Clinical Laboratories, Stanford Health Care, 3375 Hillview Ave, Palo Alto, CA 94304 USA

**Keywords:** Diagnosis, Proteins, Diagnostic markers, Bioanalytical chemistry, Mass spectrometry

## Abstract

Cerebrospinal fluid (CSF) leak can be diagnosed in clinical laboratories by detecting a diagnostic marker β_2_-transferrin (β_2_-Tf) in secretion samples. β_2_-Tf and the typical transferrin (Tf) proteoform in serum, β_1_-transferrin (β_1_-Tf), are Tf glycoforms. An innovative affinity capture technique for sample preparation, called microprobe-capture in-emitter elution (MPIE), was incorporated with high-resolution mass spectrometry (HR-MS) to study the Tf glycoforms and the primary structures of β_1_-Tf and β_2_-Tf. To implement MPIE, an analyte is first captured on the surface of a microprobe, and subsequently eluted from the microprobe inside an electrospray emitter. The capture process is monitored in real-time via next-generation biolayer interferometry (BLI). When electrospray is established from the emitter to a mass spectrometer, the analyte is immediately ionized via electrospray ionization (ESI) for HR-MS analysis. Serum, CSF, and secretion samples were analyzed using MPIE-ESI-MS. Based on the MPIE-ESI-MS results, the primary structures of β_1_-Tf and β_2_-Tf were elucidated. As Tf glycoforms, β_1_-Tf and β_2_-Tf share the amino acid sequence but contain varying N-glycans: (1) β_1_-Tf, the major serum-type Tf, has two G2S2 N-glycans on Asn413 and Asn611; and (2) β_2_-Tf, the major brain-type Tf, has an M5 N-glycan on Asn413 and a G0FB N-glycan on Asn611. The resolving power of the innovative MPIE-ESI-MS method was demonstrated in the study of β_2_-Tf as well as β_1_-Tf. Knowing the N-glycan structures on β_2_-Tf allows for the design of more novel test methods for β_2_-Tf in the future.

## Introduction

Cerebrospinal fluid (CSF) leak can occur as a result of laceration, blunt trauma, or surgery. It may lead to potentially life-threatening meningitis if left untreated^1,2^. CSF leak can be diagnosed in clinical laboratories by detecting a diagnostic marker β_2_-transferrin (β_2_-Tf) in any body fluid—most commonly in rhinorrhea or otorrhea secretion samples. β_2_-Tf is a proteoform of human transferrin (Tf) that is mainly present in CSF and barely detectable in other body fluids^3,4^. The clinical utility and diagnostic value of β_2_-Tf in CSF leak have been demonstrated^5,6^. β_2_-Tf, together with the typical Tf proteoform in serum, β_1_-transferrin (β_1_-Tf), were named after their electrophoretic mobility in gel electrophoresis^7^. While β_2_-Tf is widely used as a diagnostic marker for CSF leak, the primary structure of β_2_-Tf as well as that of β_1_-Tf have not been elucidated.

There has been extensive basic research on human Tf since it is a high-abundance protein in blood with a major role in iron metabolism. The amino acid sequence of Tf precursor was determined through protein cleavage and cDNA characterization, showing a full sequence of 698 amino acids; after the removal of an N-terminal signal peptide, the mature form of Tf contains 679 amino acids, 19 intramolecular disulfide bonds formed between cysteine residues, and two N-glycans attached to the amino groups of the side chains of Asn419 and Asn611 (Asn432 and Asn630 of Tf precursor)^8–11^. Tf proteoforms typically vary by the N-glycan structures^11–13^, and the Tf proteoforms of interest so far are Tf glycoforms, including β_1_-Tf and β_2_-Tf^7^.

Although it is known that β_2_-Tf has desialylated N-glycans while β_1_-Tf has fully sialylated N-glycans^3,14,15^, the primary structures of β_1_-Tf and β_2_-Tf have not been clarified. On the other hand, the N-glycans on Tf glycoforms in serum and in CSF were characterized using gel electrophoresis, liquid chromatography, and enzymatic digestion-based mass spectrometry (MS) by neurobiologists^13,16–19^. It was reported that a group of Tf glycoforms were present in serum, namely serum-type Tf glycoforms or sTf, among which a specific Tf glycoform (major serum-type Tf) predominated^12,13^; the serum-type Tf glycoforms also existed in CSF. An additional group of Tf glycoforms were present in CSF, namely brain-type Tf glycoforms, among which a specific Tf glycoform (major brain-type Tf) was more abundant than the rest^15,20^. The brain-type Tf glycoforms was at least partly synthesized in the CSF-producing tissue choroid plexus rather than being produced by glycosidase digestion of serum-type Tf glycoforms^16,20^. It was found that the N-glycans on the major serum-type Tf consisted of bi-antennary oligosaccharide chains with sialylated terminals, and those on the brain-type Tf glycoforms were desialylated, or more specifically, unsialylated (asialotransferrin)^15,16,18,20^. It was hypothesized that the major brain-type Tf was β_2_-Tf^21^, but this hypothesis has not been proved. Similarly, it is reasonable to presume that the major serum-type Tf was β_1_-Tf, however a proof is required.

In clinical cases where CSF leak is suspected, secretion samples can be collected from patients and sent to clinical laboratories to test for the presence of β_2_-Tf. The conventional method to test β_2_-Tf as well as β_1_-Tf is agarose gel immunofixation electrophoresis (IFE)^2–4^. Although it is widely used in clinical laboratories, it does not provide structural information of the analytes. Thus, the primary structures of β_1_-Tf and β_2_-Tf, particularly the N-glycan structures on these Tf glycoforms, remained an unanswered question with this method.

As an emerging technology in clinical diagnostics, high-resolution mass spectrometry (HR-MS), particularly top-down HR-MS, can be used to analyze a protein target in its intact state and identify the post-translational modifications and amino acid variations in its proteoforms^22–24^. While HR-MS is an ideal tool to study the Tf glycoforms, the quality of data acquired during HR-MS analysis depends on sample preparation^25,26^. In this article, an innovative affinity capture technique for sample preparation, called microprobe-capture in-emitter elution (MPIE), was incorporated with HR-MS to study the Tf glycoforms^27^. MPIE can directly couple a label-free optical sensing technology with MS. The label-free optical sensing technology is next-generation biolayer interferometry (BLI, also named as thin-layer interferometry, TFI), which senses optical thickness changes on the sensing surface of a microprobe caused by biomolecular interactions such as antibody-antigen binding, achieving real-time measurement without employing a reporter molecule (enzyme, fluorophore, etc.)^28,29^. To implement MPIE, an analyte is first captured on the surface of a microprobe, and subsequently eluted from the microprobe inside an electrospray emitter. The capture process is monitored in real-time via BLI. When electrospray is established from the emitter to a mass spectrometer, the analyte is immediately ionized via electrospray ionization (ESI) for HR-MS analysis. By this means, BLI and HR-MS are directly coupled in the form of MPIE-ESI-MS, which is readily deployed to study the Tf glycoforms and the primary structures of β_1_-Tf and β_2_-Tf.

## Methods

### Materials and specimens

LC–MS grade water, acetonitrile, formic acid, and 0.2 µm PVDF syringe filters were purchased from Thermo Fisher Scientific (Waltham, MA). A mouse monoclonal anti-transferrin IgG antibody (anti-Tf Ab) was obtained from Sinobiological (Wayne, PA), and biotinylated using an EZ-Link HPDP-Biotin reagent kit (Waltham, MA). Remnant CSF and serum samples from patients, and secretion samples from patients suspected of CSF leak were obtained from Stanford Health Care and Stanford Children’s Health, following an approved institutional review board protocol for the use of remnant patient specimens.

### Sample preparation

The biotinylated anti-Tf Ab was diluted in phosphate-buffered saline at pH 7.4 with 0.02% Tween 20 and 0.2% BSA (PBST-B) to 10 μg/ml for use. A pooled CSF sample was made by mixing 9 CSF samples from patients to explore the analytical sensitivity of MPIE-ESI-MS for β_2_-Tf in CSF. The pooled CSF sample was mixed with water to make a dilution series. CSF samples were 1:1 diluted in phosphate-buffered saline at pH 7.4 with 0.02% Tween 20 (PBST) and serum samples were 1:19 diluted in PBST-B. Secretion samples from patients were first mixed with an equal amount of water and filtered using a 0.2 µm PVDF syringe filter, and then 1:1 diluted in PBST.

To study the gel electrophoresis-separated Tf glycoforms, gel electrophoresis of CSF samples was carried out using a Hydragel 6 β_2_ Transferrin kit (Sebia, Lisses, France) following the manufacturer’s protocol. In brief, a CSF sample was first 1:1 mixed with an iron-saturating solution, and then 10 µl of the sample was loaded to each of the 6 wells on an agarose gel so that replicates were run in all the 6 lanes. Gel electrophoresis was implemented in a Hydrasys 2 instrument (Sebia, Lisses, France), and the agarose gel was removed from the instrument immediately after the gel electrophoresis was completed, without carrying out the immunofixation steps. The agarose gel was placed on a paper template marked with the β_1_-Tf and β_2_-Tf band regions, and the gel stripes of the band regions were cut out using a scalpel. Each gel stripe was placed in a 1.5 ml sample tube, 200 µl PBST was added, and the sample was rocked for 2 h at room temperature to extract the analyte from the gel stripe. After extraction, the supernatant was filtered using a 0.2 µm PVDF syringe filter. The images of an agarose gel for MPIE-ESI-MS analysis and a reference agarose gel after immunofixation are posted in Fig. 2.

### MPIE-ESI-MS instrumentation and experiment

An MPIE-ESI-MS experiment consists of two parts: BLI-based affinity capture and in-emitter elution ESI-MS, with the details including instrumentation described elsewhere^27^. The BLI-based affinity capture was implemented in a Gator Plus analyzer (Gator Bio, Palo Alto, CA): a BLI microprobe pre-coated with streptavidin was first dipped into the biotinylated anti-Tf Ab solution for 10 min to load the anti-Tf Ab, then dipped into a sample for 10 min to capture Tf molecules, and rinsed in PBST for 1 min to remove non-specifically bound molecules. The in-emitter elution ESI-MS was implemented in an EMASS-II ESI ion source which coupled an ECE-001 capillary electrophoresis instrument (CMP Scientific, Brooklyn, NY) with an Orbitrap Q-Exactive Plus mass spectrometer (Thermo Scientific, San Jose, CA): an electrospray emitter with a regular open end and a tapered open end (tip orifice diameter 20–30 μm) was filled with a sheath liquid (10 mM ammonium formate in water); after affinity capture, the microprobe was rinsed in the sheath liquid for 10 s, inserted into the emitter through the regular open end, and settled in the tapered end by gravity; the emitter was mounted to the ESI ion source, and a capillary was inserted into the emitter through the regular open end and positioned right behind the microprobe to deliver an elution liquid (80% acetonitrile and 2% formic acid in water); once electrospray was established by applying a positive voltage to the sheath liquid in the emitter, HR-MS data acquisition was initiated, and injection of the elution liquid was started subsequently. The emitter was placed ~ 2 mm away from the mass spectrometer inlet with the electrospray voltage set at 2.2 kV. The injection of the elution liquid was driven by 5 psi pneumatic pressure. The following MS parameters were used: ion-transfer capillary temperature 350 °C, S-lens RF level 50, and number of microscans 10. Primary mass spectra were acquired in positive polarity at resolution 17.5 K.

### Data analysis

In HR-MS analysis of proteins, it is necessary to deconvolute raw MS data to merge the multiple charge states and isotopic peaks of an analyte to obtain its accurate molecular mass. The acquired data in each MPIE-ESI-MS experiment was viewed as a time trace of MS responses, and the elution time window of an analyte was identified by checking the molecular ions of the analyte at each time point. The data in the elution time window were selected for deconvolution using Biopharma Finder 4.1 (Thermo Fisher Scientific, San Jose, CA) with the ReSpect algorithm. MS peaks of analytes were displayed in deconvoluted mass spectra at uncharged state showing average molecular masses.

### Ethics approval

Informed consent was not required because only remnant clinical samples were used in this study. An institutional review board approval (Protocol 66,075 by Panel on Medical Human Subjects of Stanford University) for the use of remnant patient specimens was obtained. All methods were carried out in accordance with relevant guidelines and regulations.

## Results

**Figure 1:**
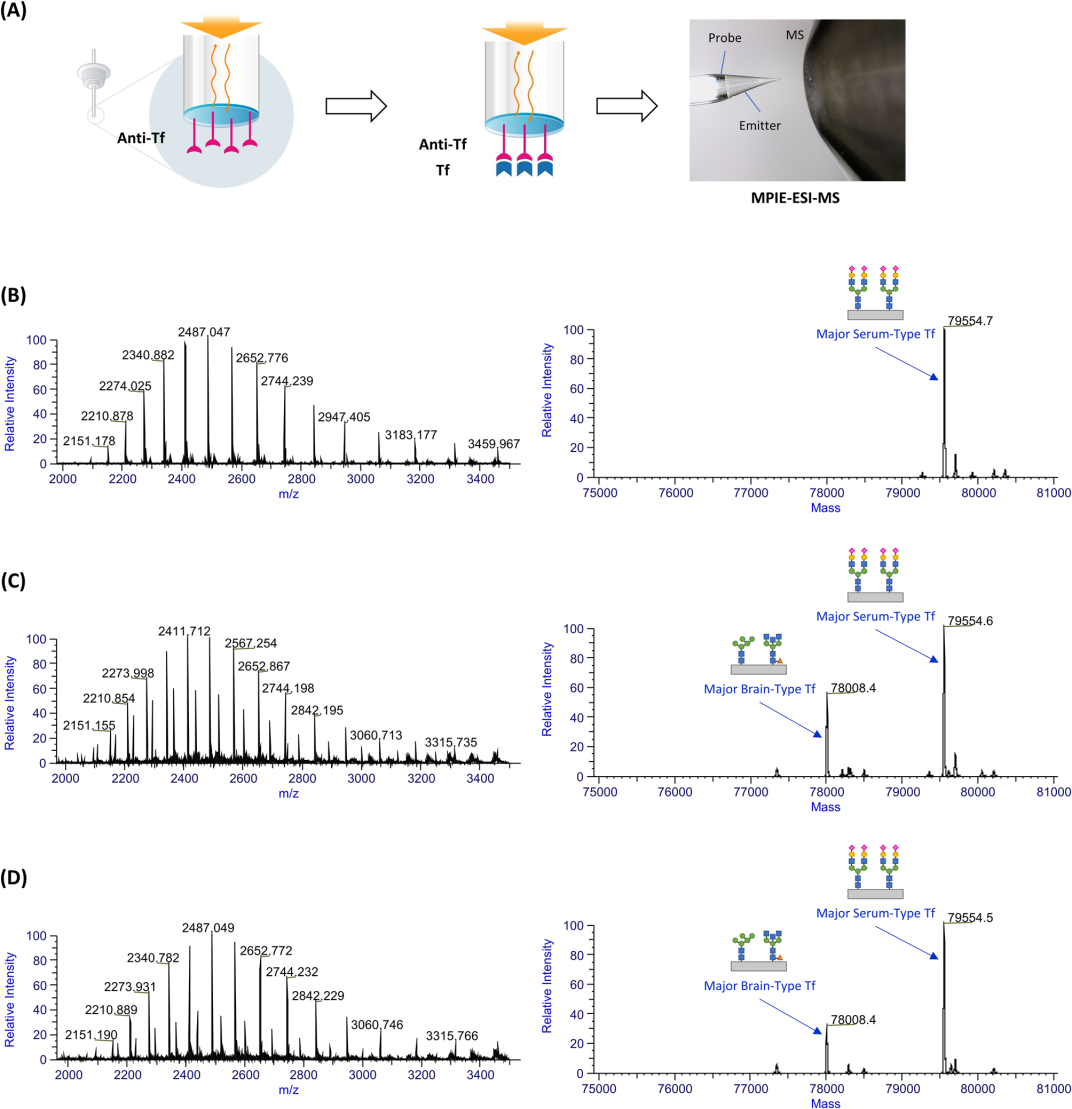
(**A**) Experiment workflow of MPIE-ESI-MS for Tf analysis. The MPIE-ESI-MS results of (**B**) a serum sample, (**C**) a CSF sample, and (**D**) a secretion sample from a patient diagnosed of CSF leak: HR-MS raw mass spectra (left) and deconvoluted mass spectra (right) of captured Tf molecules, showing serum-type Tf glycoforms in (**B**), and both serum-type Tf and brain-type Tf glycoforms in (**C**) and (**D**).

The performance of MPIE-ESI-MS for Tf analysis was demonstrated and reported elsewhere^27^. MPIE-ESI-MS had a limit of detection for the Tf standard at 0.063 μg/ml, which was translated to no more than 7 fmol Tf molecules captured on a microprobe^27^. The high analytical sensitivity and specificity provided by affinity capture made MPIE-ESI-MS an ideal method to study the Tf molecules. The results of a set of serum, CSF, and secretion samples are shown in Fig. 1. The deconvoluted mass spectrum of the serum sample showed a group of MS peaks around 79,554 Da; they are mainly serum-type Tf glycoforms and the predominant MS peak at 79,554 Da is the major serum-type Tf (N-glycan structures shown in Fig. 1, see details in Discussion). The deconvoluted mass spectrum of the CSF sample showed the major serum-type Tf and a group of MS peaks around 78,008 Da; they are mainly brain-type Tf glycoforms and the most abundant MS peak at 78,008 Da is the major brain-type Tf (N-glycan structures shown in Fig. 1, see details in Discussion). The deconvoluted mass spectrum of the secretion sample showed both serum-type and brain-type Tf glycoforms, meaning that CSF was present in the sample. This finding was consistent with the fact that the secretion sample was obtained from a patient diagnosed with CSF leak. In addition, the BLI sensorgrams (a time trace of label-free optical sensing responses) during the capture of Tf as well as the BLI sensorgrams of negative controls (PBST-B and PBST) are shown in Supplementary Figure S1. The BLI measurement facilitates real-time monitoring of the capture process.

**Figure 2:**
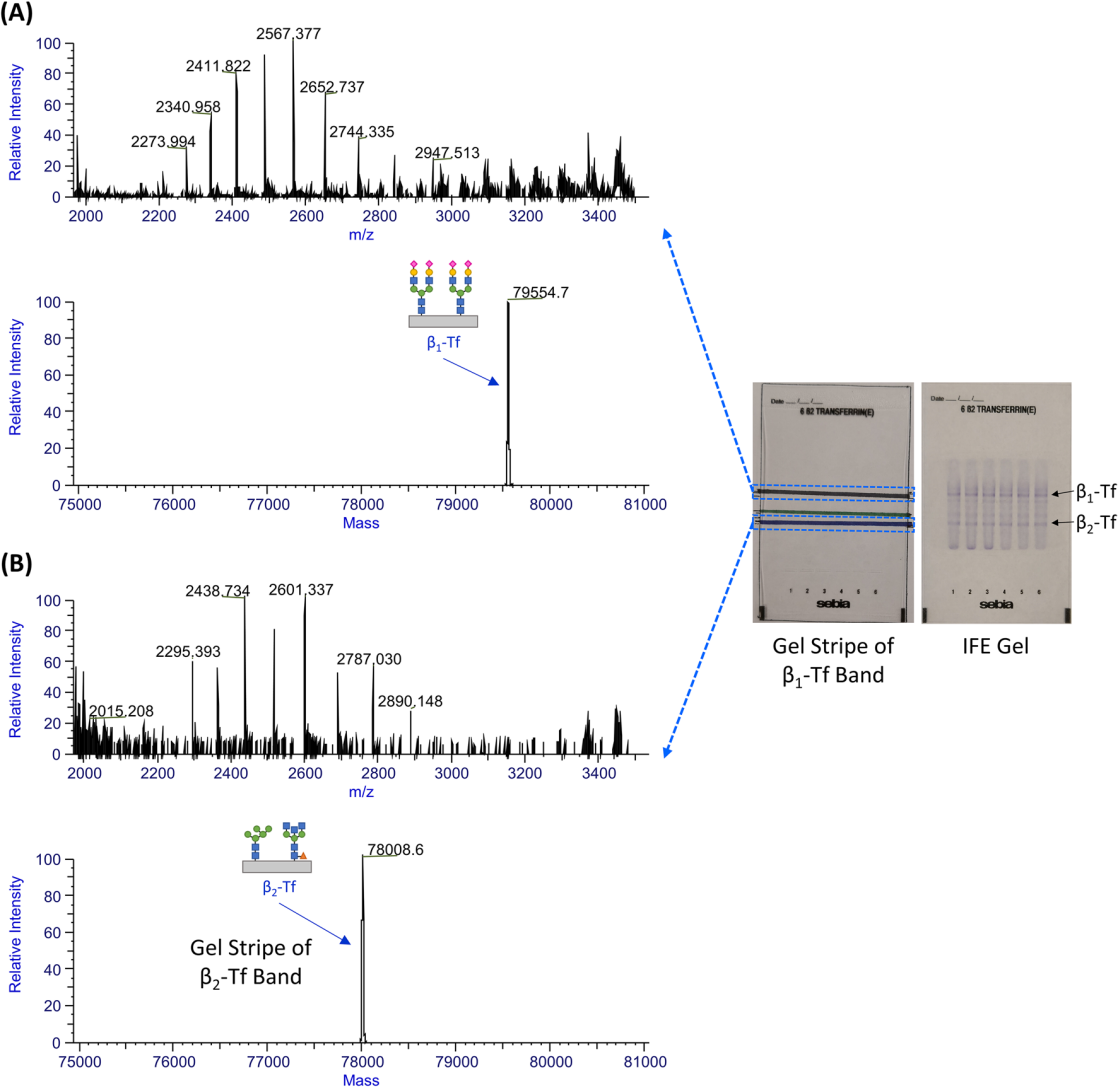
The MPIE-ESI-MS results of (**A**) the extract from the gel stripe of the β_1_-Tf band region and (**B**) the extract from the gel stripe of the β_2_-Tf band region: HR-MS raw mass spectra (left) and deconvoluted mass spectra (right) of captured Tf molecules, showing β_1_-Tf in (**A**) and β_2_-Tf in (**B**). The image of an agarose gel after gel electrophoresis is posted to the right of the mass spectra, which was placed on the paper template marked with the β_1_-Tf and β_2_-Tf band regions. The image of a reference agarose gel after immunofixation is posted further on the right, indicating the β_1_-Tf and β_2_-Tf band regions designated according to the manufacturer’s protocol.

To figure out the relationship between the major serum-type Tf, the major brain-type Tf and β_1_-Tf, β_2_-Tf, after gel electrophoresis of CSF samples, the extracts from the gel stripes of the β_1_-Tf and β_2_-Tf band regions were analyzed using MPIE-ESI-MS. The results of a CSF sample are shown in Fig. 2. The deconvoluted mass spectra showed only the MS peak of the major serum-type Tf in the extract from the β_1_-Tf band region and only the MS peak of the major brain-type Tf in the extract from the β_2_-Tf band region. The measured accurate molecular masses were consistent with the experiments in Fig. 1 and matched the theoretical molecular masses of the major serum-type Tf and major brain-type Tf (N-glycan structures shown in Fig. 2, see details in Discussion). This observation proved the hypothesis that β_1_-Tf and β_2_-Tf were actually the major serum-type Tf and major brain-type Tf, respectively. In addition, the gel area between the β_1_-Tf and β_2_-Tf band regions was also analyzed and minor Tf glycoforms in serum and CSF were not found, which could be due to the low quantities of the minor Tf glycoforms in a gel stripe.

A collection of 11 secretion samples from patients suspected of CSF leak were analyzed using the MPIE-ESI-MS method, among which 5 samples were positive for β_2_-Tf and the rest were negative as measured by the conventional IFE test. As shown in Table 1, the MS peak at 78,008 Da was observed in the MPIE-ESI-MS results of the 5 positive samples but not found in those of the 6 negative samples, which confirmed the consistency between the MPIE-ESI-MS method and the conventional IFE test. In addition, the limit of detection of the MPIE-ESI-MS method was explored. A pooled CSF sample was mixed with water at 1:1, 1:4, 1:9, and 1:19 ratios to prepare a dilution series for analysis. Supplementary Table S1 summarizes the results, listing the MS peak intensities of β_1_-Tf and β_2_-Tf in the deconvoluted mass spectra (the entire time window of Tf elution selected for deconvolution). The peak intensities decreased with the pooled CSF sample dilution, and it was demonstrated that the MPIE-ESI-MS method was able to detect β_2_-Tf in at least tenfold diluted CSF (1:9 pooled CSF: water mixture).

**Table 1:** The MPIE-ESI-MS results of 11 secretion samples from patients suspected of CSF leak.

Secretion sample	β1-Tf		β2-Tf	
	Detected?	Measured molecular mass	Detected?	Measured molecular mass
Positive sample 1	Yes	79,554.50 Da	Yes	78,008.42 Da
Positive sample 2	Yes	79,554.79 Da	Yes	78,008.60 Da
Positive sample 3	Yes	79,554.75 Da	Yes	78,008.83 Da
Positive sample 4	Yes	79,554.04 Da	Yes	78,008.68 Da
Positive sample 5	Yes	79,554.77 Da	Yes	78,008.03 Da
Negative sample 1	Yes	79,554.29 Da	No	/
Negative sample 2	Yes	79,554.80 Da	No	/
Negative sample 3	Yes	79,554.81 Da	No	/
Negative sample 4	No	/	No	/
Negative sample 5	Yes	79,554.49 Da	No	/
Negative sample 6	Yes	79,554.73 Da	No	/

## Discussion

**Figure 3:**
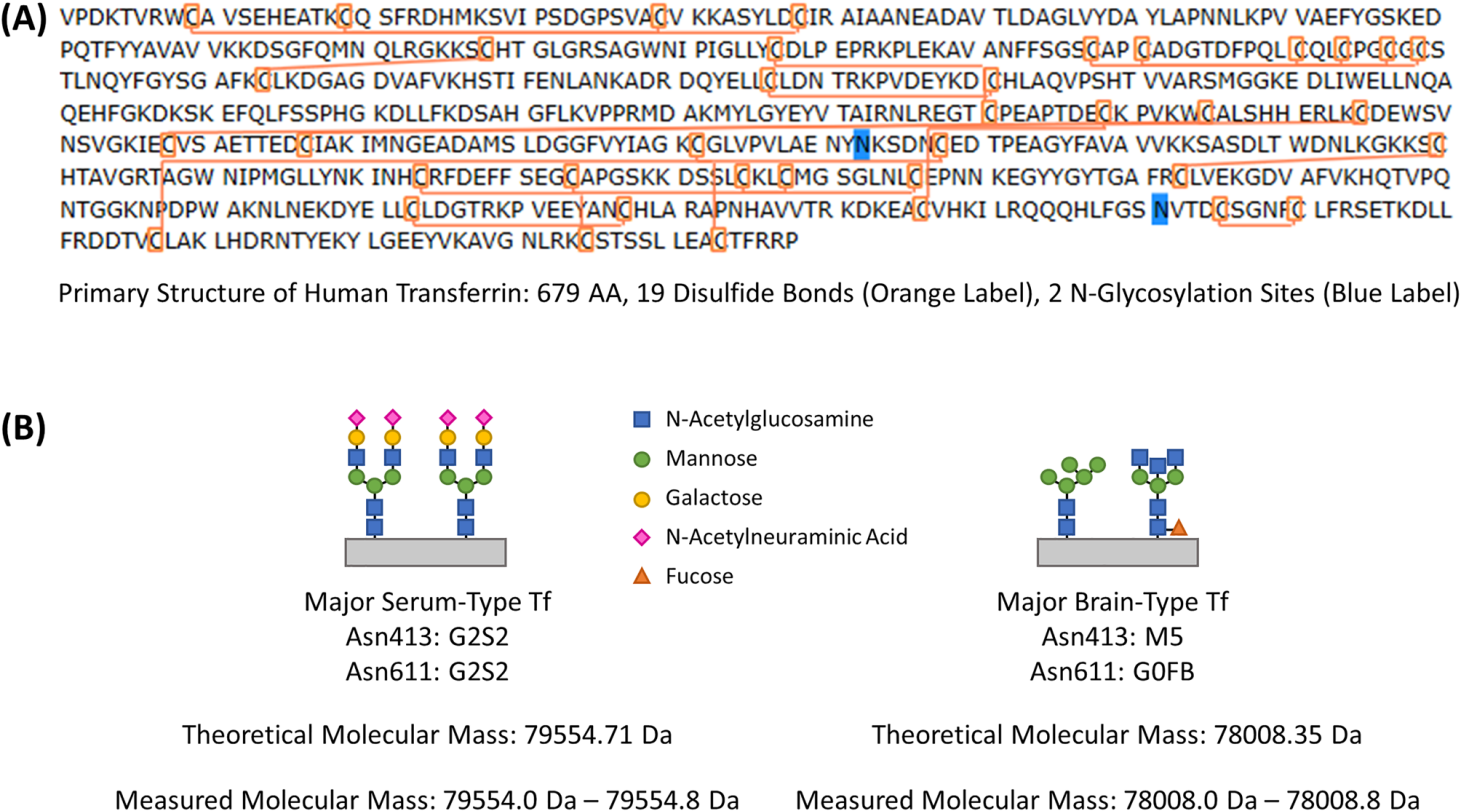
(**A**) Primary structure of human transferrin, showing the sequence of 679 amino acids, 19 disulfide bonds, and 2 N-glycosylation sites. (**B**) N-glycan structures on β_1_-Tf (major serum-type Tf) and β_2_-Tf (major brain-type Tf), confirmed by comparing the theoretical molecular masses of the Tf glycoforms with the measured molecular masses in Figs. 1, 2, and Table 1.

The MPIE-ESI-MS analysis of the extracts from the gel stripes proved that β_1_-Tf and β_2_-Tf were identical to the major serum-type Tf and the major brain-type Tf, respectively. As Tf glycoforms, β_1_-Tf and β_2_-Tf share the amino acid backbone but contain varying N-glycans. The amino acid sequence of Tf was previously investigated and reported^8,9^. The primary structure of a mature Tf molecule is composed of 679 amino acids with 19 disulfide bonds and 2 N-glycans, as illustrated in Fig. 3A. N-glycans on proteins typically include bi-, tri-, or tetra-antennary oligosaccharide chains resulted from sequential action of glycosyltransferases^30,31^, and an N-glycan can be named according to its sugar composition and branching structure (traditional nomenclature used in this article)^32^. When the potential varieties of N-glycans are established, the structures of Tf glycoforms can be confirmed by aligning the measured accurate molecular masses with the theoretical molecular masses of the potential Tf glycoforms.

The potential structures of N-glycans on Tf were previously reported by employing enzymatic digestion-based MS analysis of the Tf glycoforms in CSF^17,19^. It was revealed that the most abundant N-glycan type was G2S2. Quantitation of the digested glycopeptides encompassing the glycosylation sites found that G2S2 consisted of roughly 65% of all N-glycans on Asn413 and 73% of all N-glycans on Asn611^17^. Thus, the Tf glycoform with two G2S2 N-glycans on Asn413 and Asn611 aligns with β_1_-Tf for the following reasons: (1) the measured molecular mass of β_1_-Tf exactly matches its theoretical molecular mass 79,554.71 Da (Fig. 3B), (2) it is the most abundant Tf glycoform in serum and CSF, and (3) it is consistent with the fact that β_1_-Tf is fully sialylated (N-acetylneuraminic acid as the sialic acid). In addition, the Tf glycoforms associated with M5 and G0FB N-glycans must be the most abundant unsialylated Tf glycoforms in CSF provided that the two N-glycans were found to be the most abundant unsialylated N-glycans^19^. Particularly, M5 was found consisting of roughly 20% of all N-glycans on Asn413 and G0FB was found consisting of 26% of all N-glycans on Asn611^17^. Among the possible combinations of the two N-glycans, the Tf glycoform with an M5 N-glycan and a G0FB N-glycan matches β_2_-Tf because (1) the measured molecular mass of β_2_-Tf exactly matches its theoretical molecular mass 78,008.35 Da (Fig. 3B), and (2) it is consistent with the fact that β_2_-Tf is unsialylated. The glycosylation sites of the M5 and G0FB N-glycans were found to be on Asn413 and Asn611 respectively by another study of digested glycopeptides from Tf glycoforms in CSF^18^. The presence of a bisecting N-acetylglucosamine (GlcNAc) in the G0FB N-glycan was proved by the previously reported lectin-binding experiments^16^, verifying the structure of G0FB N-glycan over another possible isomeric N-glycan without a bisecting GlcNAc^19^. Thus, the analysis above substantiates the following findings: (1) β_1_-Tf, the major serum-type Tf, has two G2S2 N-glycans on Asn413 and Asn611; and (2) β_2_-Tf, the major brain-type Tf, has an M5 N-glycan on Asn413 and a G0FB N-glycan on Asn611.

Besides the two major Tf glycoforms in CSF (β_1_-Tf and β_2_-Tf), it is known that other minor Tf glycoforms may also exist in CSF, blood, and other body fluids^4,33^. As illustrated in the previous literature, there are a variety of unsialylated N-glycans on Tf molecules, and the sialylated N-glycan G2S2 also has diversity such as the more branched form G3S3^13,17,34^. Thus, a number of fully sialylated, partially sialylated, and unsialylated Tf glycoforms can be formed by the various combinations of the two N-glycans on a Tf molecule. In gel electrophoresis, sialic acids bring negative charges to a Tf molecule under neutral or alkaline pH conditions, influencing its electrophoretic mobility. As such, Tf glycoforms migrate in the order of fully sialylated, disialylated, and unsialylated Tf glycoforms, with regard to the number of sialic acids on the N-glycans. Thus, the minor Tf glycoforms can migrate within or between the β_1_-Tf and β_2_-Tf band regions. For instance, the product insert of the Hydragel 6 β_2_ Transferrin kit states that a band of disialylated Tf glycoforms (disialotransferrin) may exist above the β_2_-Tf band^35^. On the other hand, the unsialylated Tf glycoforms besides β_2_-Tf can migrate within the β_2_-Tf band region and interfere with the β_2_-Tf detection. The product insert suggests to use the ratio of unsialylated to disialylated bands to confirm the presence of β_2_-Tf. The reason of this practice can be explained as follows: when CSF is present in a sample, the abundance of the major unsialylated Tf glycoform β_2_-Tf significantly exceeds the minor unsialylated and disialylated Tf glycoforms, resulting in a high ratio of the β_2_-Tf band to the disialotransferrin band. This practice in the IFE test can be achieved in the MPIE-ESI-MS analysis as all the Tf glycoforms are shown in a deconvoluted mass spectrum and the MS peak intensities represent their relative quantities. However, it is probably unnecessary because β_2_-Tf can be specifically detected by its accurate molecular mass and definitively differentiated from other unsialylated and disialylated Tf glycoforms.

The demonstration of MPIE-ESI-MS in detection of β_2_-Tf paved a way to establish an MS-based clinical assay for β_2_-Tf. When implementing the accurate molecular mass-based detection of β_2_-Tf, Tf variants resulted from genetic polymorphism should be taken into consideration^11,36–39^. In theory, amino acid variation in the Tf molecule can change the molecular masses of β_1_-Tf and β_2_-Tf but not the molecular mass difference between the two Tf glycoforms (1546 Da), provided the two glycosylation sites are not modified. This hypothesis is supported by the MPIE-ESI-MS results of a few Tf variant-containing CSF samples, as shown in Supplementary Figure S2A and S2B. In addition, the N-glycans on serum-type Tf glycoforms can vary under specific pathophysiological conditions such as carbohydrate-deficient syndromes and alcohol abuse. β_1_-Tf might not be found in those samples due to aberrant glycosylation^12,34,40–42^. As an example, Supplementary Figure S2C shows altered Tf glycoforms in a CSF sample from an alcohol-consuming patient. These topics are beyond the scope of this article and can be investigated in the future when an MS-based clinical assay for β_2_-Tf is being developed.

As an innovative affinity capture technique, MPIE facilitates real-time monitoring of the affinity capture process to overcome the lack of process monitoring in conventional affinity capture techniques. The assembly of a BLI microprobe and an electrospray emitter restricts dispersion of eluted analyte in MPIE-ESI-MS and substantially brings up the concentration of the minute amount of analyte captured by a BLI microprobe, allowing for good analytical sensitivity. Currently MPIE-ESI-MS is not an ideal tool for quantitative analysis due to the variation in capture agent loading and analyte binding on a BLI microprobe, as well as that in MS ionization. In the future, when accurate quantitation is needed, quantitative MPIE-ESI-MS analysis should be achievable by employing internal standards, i.e., preferably stable isotope-labeled analytes^27^.

## Conclusions

The resolving power of the innovative MPIE-ESI-MS method was demonstrated in the study of β_2_-Tf as well as β_1_-Tf. Knowing the N-glycan structures on β_2_-Tf allows for the design of more novel test methods for β_2_-Tf in the future. For instance, it is possible to employ specific carbohydrate-binding lectins to selectively capture β_2_-Tf for MS analysis or lectin-antibody assays.

### Supplementary Information

Supplementary Information.

## Data Availability

All data generated or analyzed during this study are included in this published article and its supplementary information.

## Abbreviations

CSF: Cerebrospinal fluid
Tf: Transferrin
β_1_-Tf: β_1_-transferrin
β_2_-Tf: β_2_-transferrin
MPIE: Microprobe-capture in-emitter elution
ESI: Electrospray ionization
HR-MS: High-resolution mass spectrometry
BLI: Biolayer interferometry
IFE: Immunofixation electrophoresis
