# Supplementary material for: Study of β1-transferrin and β2-transferrin using microprobe-capture in-emitter elution and high-resolution mass spectrometry
Source: Sci Rep. 2023 Sep 11;13:14974. doi: 10.1038/s41598-023-42064-7 (PMC10495423; doi:10.1038/s41598-023-42064-7)
Supplement: Supplementary file 1 — Supplementary Information. [file 41598_2023_42064_MOESM1_ESM.pdf]

Supplementary Information

Study of β_1-Transferrin and β_2-Transferrin Using Microprobe-Capture In-Emitter Elution and High-Resolution Mass Spectrometry

Ruben Yiqi Luo[1,2], Christopher Pfaffroth[2], Samuel Yang[1], Kevin Hoang[2], Priscilla S.-W. Yeung[1,2], James L. Zehnder[1,2], Run-Zhang Shi[1,2]

[1]Department of Pathology, Stanford University, Stanford, CA, USA

[2]Clinical Laboratories, Stanford Health Care, Palo Alto, CA, USA

Running Title: Primary Structure Elucidation of β_2-Transferrin

Corresponding Author: Ruben Yiqi Luo

Address: 3375 Hillview Ave, Palo Alto, CA 94304

Tel: 650-724-1318

Email: rubenluo@stanford.edu

Key Words: β_1-Transferrin, β_2-Transferrin, MPIE, HR-MS, N-Glycan

Supplementary Figures

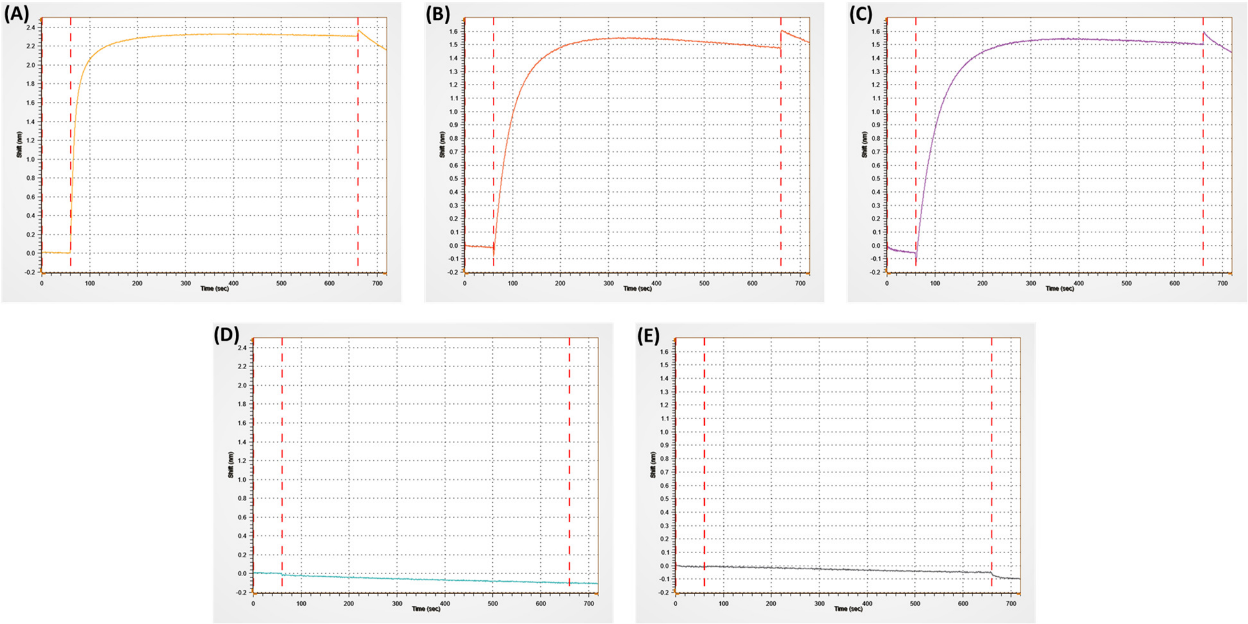

Figure S1. BLI sensorgrams obtained on the 3 BLI microprobes capturing Tf from a serum sample (A), a CSF sample (B), and a secretion sample from a patient diagnosed of CSF leak (C) (same samples as in Figure 1), and BLI sensorgrams obtained on the 2 BLI microprobes in PBST-B (D) and PBST (E) as negative controls. The BLI signal “jump” between the capture step and the rinse step was caused by refractive index difference between the sample and the buffer (PBST-B for serum samples, PBST for CSF and secretion samples).

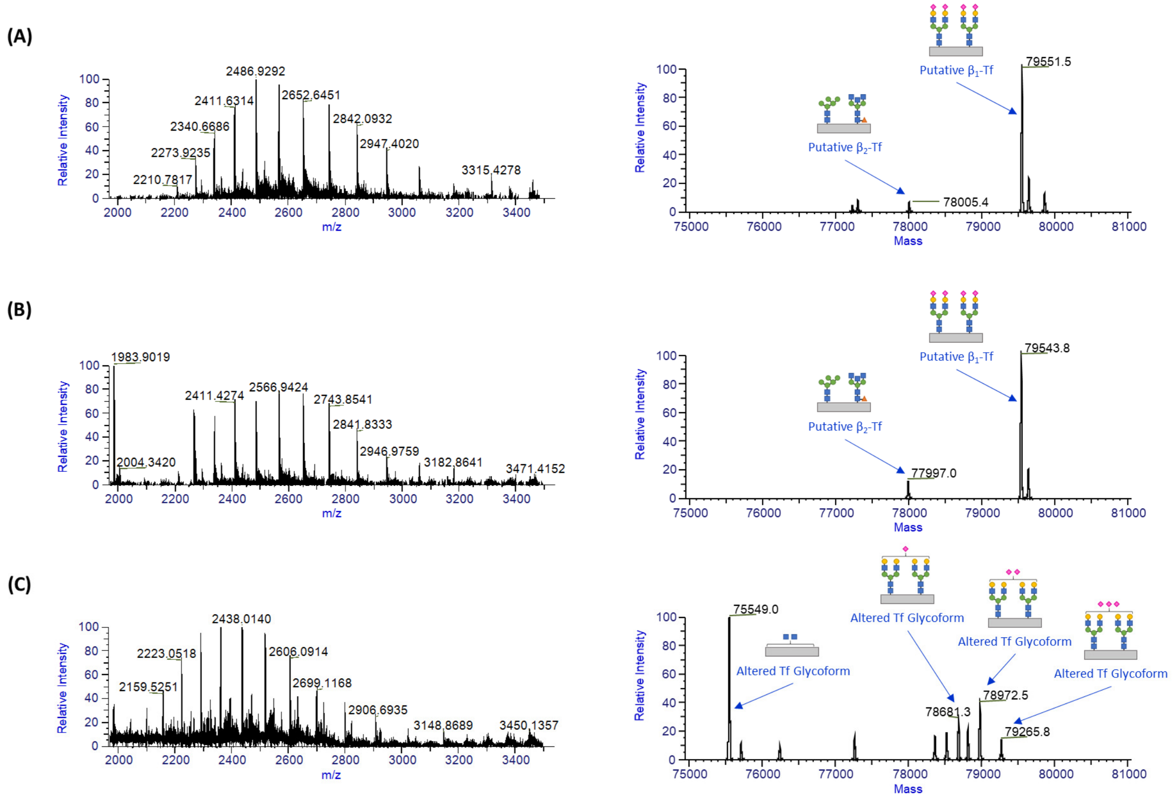

Figure S2. (A) and (B) The MPIE-ESI-MS results of two CSF samples containing Tf variants: HR-MS raw mass spectra of captured Tf molecules (left) and deconvoluted mass spectra (right), showing the Tf glycoforms. The molecular masses of the putative β_1-Tf and β_2-Tf shifted a same value from those of β_1-Tf and β_2-Tf in normal CSF samples, while the mass difference between the two Tf glycoforms retained as 1546 Da, indicating that the mass shift was caused by amino acid variation in the Tf molecule. (C) The MPIE-ESI-MS result of a CSF sample from an alcohol-consuming patient, showing MS peaks of altered Tf glycoforms that are labeled with presumptive N-glycan structures inferred from the intact molecular masses.

Supplementary Tables

Table S1. MS peak intensities of β_1-Tf and β_2-Tf in the deconvoluted mass spectra (the entire time window of Tf elution selected for deconvolution): a pooled CSF sample was mixed with water at 1:1, 1:4, 1:9, and 1:19 ratios, the neat pooled CSF sample, and the pooled CSF sample spiked with 10 µg/ml and 100 µg/ml Tf standard.

Sample	MS Peak Intensity of β1-Tf	MS Peak Intensity of β2-Tf
1:1 Pooled CSF : Water	4.10×10^5	1.62×10^5
1:4 Pooled CSF : Water	2.52×10^5	7.03×10^4
1:9 Pooled CSF : Water	9.47×10^4	1.98×10^4
1:19 Pooled CSF : Water	4.18×10^4	Not Detected
